# From the archives: A plant immune hub before, after, and *way* after its discovery

**DOI:** 10.1093/plcell/koae205

**Published:** 2024-07-24

**Authors:** Bradley Laflamme

**Affiliations:** Assistant Features Editor, The Plant Cell, American Society of Plant Biologists; Department of Molecular Genetics, University of Toronto, Toronto, ON M5S 1A1, Canada

## February 1996: Not so fast: an epistatic interaction between effector proteins leads to immune suppression

**Figure 1.**
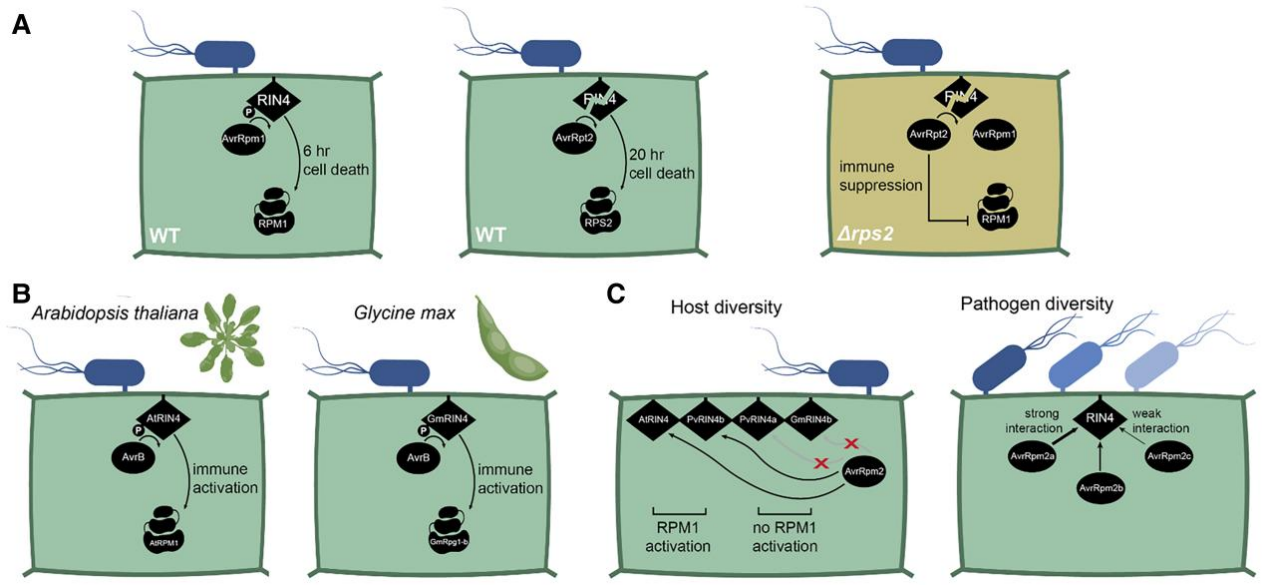
Effector perturbations to RIN4 in immunity across the ages. **A)** An epistatic interaction between AvrRpt2 and AvrRpm1 suppresses immunity. Either effector in isolation triggers RPM1- or RPS2-dependent immunity at a specific time point through their distinct modifications of the host immune hub RIN4. When both effectors are coexpressed in an *Δrps2* mutant, RIN4 cleavage by AvrRpt2 suppresses AvrRpm1-triggered immunity. **B)** Convergent evolution across plant lineages to perceive bacterial perturbations: AvrB-induced modifications to RIN4 lead to activation of RPM1 in *Arabidopsis thaliana*, while they lead to activation of a convergently evolved receptor, Rpg1-b, in soybean (*Glycine max*). **C)** Host and pathogen diversity both influence immune hub activation. Host diversity: AvrRpm2 modifies specific allelic variants of RIN4 (AtRIN4 and PvRIN4b) in a manner that activates immunity. Pathogen diversity: alleles of AvrRpm2 from distinct pathogenic strains of *Pseudomonas syringae* (represented by different shades of blue) have quantitatively distinct effects on RIN4 activation and associated immune outputs. Figure credit: Tamar Av-Shalom.

Many plant pathogenic microbes deploy a repertoire of effector proteins to dampen host immunity and facilitate virulence, and these same effectors can be recognized by host receptors to activate immunity (Jones et al. 2024). These days, we take for granted that different effectors within these large repertoires can interact with one another—either physically or genetically—to influence host–pathogen outcomes. A major reason for that assumption is a 1996 study by Ritter and Dangl (1996). The duo noted that 2 immune-eliciting effectors from the bacterial phytopathogen *Pseudomonas syringae*, AvrRpm1 and AvrRpt2, elicited a localized cell death response in *Arabidopsis thaliana* characteristic of immune activation at starkly different timepoints (∼6 hr post-inoculation for AvrRpm1, ∼20 hr for AvrRpt2), leading them to ask which timepoint would win out if the 2 effectors were simultaneously introduced. Contrary to their expectations, no cell death was observed at 6 hr when the 2 effectors were coexpressed, suggesting that AvrRpm1-triggered immunity is blocked by the presence of AvrRpt2. Further supporting this, when they knocked out RPS2, the resistance protein that recognizes AvrRpt2, *P. syringae* carrying both effectors was able to grow to wild-type levels despite the host still having RPM1. This elegant study by Ritter and Dangl was ahead of its time in considering the potential for effectors to genetically interact with one another to suppress immunity, providing a foundation for many significant studies in plant–pathogen interactions. Even more impressive, these findings helped pave the way for the discovery of the immune hub RIN4, a membrane-associated protein that is a key component of both AvrRpm1- and AvrRpt2-triggered immunity (Mackey et al. 2002, 2003), as well as a target of many other bacterial effectors (Fig. 1A). It is astonishing how many future studies into these effectors and RIN4 arose out of the simple observation that AvrRpt2 suppresses AvrRpm1-triggered immunity.

## February 2004: Divergent plant lineages share secrets in defending against pathogen effectors

The insights of Ritter and Dangl (1996) were part of a broader movement at the turn of the century to understand indirect mechanisms of plant immune activation, that is, ways in which host receptors could guard other host proteins (sometimes called “guardees”) to recognize pathogen activities and trigger immunity (Van Der Biezen and Jones 1998; Dangl and Jones 2001). Ashfield et al. (2004) performed a comparative study of immune activation in Arabidopsis and soybean (*Glycine max*), providing further evidence that divergent immune receptors might capture the same effectors through guarding conserved processes. It was known that a distinct *P. syringae* effector, AvrB, was also recognized by RPM1 in Arabidopsis (Bisgrove et al. 1994), but resistance to AvrB had only been mapped to a cluster of resistance genes in soybean. Ashfield and colleagues first identified the specific resistance gene required for AvrB recognition using fine mapping, arriving at a single locus, *Rpg1-B*. While the protein product of *Rpg1-B* was structurally similar to that of *RPM1—*a tripartite structure containing a coiled-coil, nucleotide-binding site, and leucine-rich repeat domains—sequence analysis highlighted that these 2 genes were not orthologs, meaning that RPM1 and Rpg1-B had convergently evolved to recognize AvrB. The group showed that AvrRpt2—which suppresses RPM1-triggered immunity in Arabidopsis—also suppressed Rpg1-B–triggered immunity, suggesting that AvrRpt2 may be interfering with a common signaling component in these plant species. While we now know that suppression of AvrB (or AvrRpm1) by AvrRpt2 relates to RIN4 in both Arabidopsis and soybean (Kim et al. 2005; Selote and Kachroo 2010), the authors were careful not to overinterpret their findings, noting that AvrRpt2-mediated immune suppression could be related to another virulence target of the effector(s). They nonetheless hypothesized (correctly) that distinct receptors across plant lineages might evolve to perceive modifications to the same guardee (e.g. RIN4), since guardees are likely to be high-value targets for bacterial effectors and that epistasis between effectors might occur when these high-value targets are shared between effectors. Ashfield et al. (2004) were among the first to suggest that one of the ways divergent plant lineages manage to guard against diverse pathogens is through the evolution of distinct receptors that perceive similar effector-mediated perturbations (Fig. 1B).

## December 2022: A new spin on an old favourite: AvrRpm2 interaction dynamics with RIN4

Fast-forward another 15 to 20 years and RIN4 has enjoyed a long reputation as one of the most fascinating proteins in plant immunity, broadly targeted by pathogen effectors and a key player in both immune activation and suppression. However, there is still much to understand about the molecular mechanisms underlying RIN4 activation by a range of different effectors, particularly when we consider allelic variation within protein families and how this ultimately promotes immune activation only in some cases. Work by Yoon et al. (2022) explored RIN4 activation by AvrRpm2, an effector that is related to but shares only ∼50% sequence identity with AvrRpm1. As expected, the group found that AvrRpm2 shares several features with AvrRpm1: both had the same biochemical function (namely, ADP-ribosyltransferase activity) dependent on a conserved H-Y-D motif, and both were able to modify Arabidopsis RIN4 in a way that activates RPM1. To better understand the RIN4 residues that govern immune activation by AvrRpm2, the group made use of a paired bacterial system: they first infiltrated *Nicotiana benthamiana* with *Agrobacterium tumefaciens* to transiently express 4 RIN4 homologs (Arabidopsis, soybean, and 2 snap bean alleles), then introduced *P. syringae* expressing AvrRpm2, and finally, investigated whether RIN4 was ADP-ribosylated in the presence of the effector. Remarkably, only 2 of the tested orthologs (Arabidopsis and 1 snap bean allele) were modified and able to activate RPM1. The group homed in on one RIN4 residue, E156, which correlated across homologs with the activation of RPM1 after incubation with AvrRpm2. Loss of E156 in RIN4 ablated activation of RPM1 by AvrRpm2 coexpression, suggesting that this was a key ADP-ribosylation site for the effector. In contrast, AvrB, an effector that activates RPM1 without ADP-ribosylation activity, could still activate RPM1 without RIN4 E156, revealing that RIN4 activation through distinct effector families is driven by distinct biochemical modifications. The group also investigated other allelic variants of AvrRpm2 from distinct *P. syringae* strains and found that despite sharing high sequence identity, these alleles interacted with RIN4 with variable affinities and triggered immunity to varying degrees. This study is a powerful investigation of diversity from both host and pathogen perspectives (Fig. 1C). One gets the sense that after 20-plus years of studying RIN4—directly or indirectly—we still have an enormous amount to uncover.
